# Spatial modeling of HIV and HSV-2 among women in Kenya with spatially varying coefficients

**DOI:** 10.1186/s12889-016-3022-0

**Published:** 2016-04-22

**Authors:** Elphas Okango, Henry Mwambi, Oscar Ngesa

**Affiliations:** School of Mathematics, Statistics and Computer Science, University of KwaZulu -Natal, Private Bag X01, 3201 Pietermaritzburg, South Africa; Mathematics and Informatics Department, Taita Taveta University College, P.O Box 635-80300, Voi, Kenya

## Abstract

**Background:**

Disease mapping has become popular in the field of statistics as a method to explain the spatial distribution of disease outcomes and as a tool to help design targeted intervention strategies.

Most of these models however have been implemented with assumptions that may be limiting or altogether lead to less meaningful results and hence interpretations. Some of these assumptions include the linearity, stationarity and normality assumptions. Studies have shown that the linearity assumption is not necessarily true for all covariates. Age for example has been found to have a non-linear relationship with HIV and HSV-2 prevalence. Other studies have made stationarity assumption in that one stimulus e.g. education, provokes the same response in all the regions under study and this is also quite restrictive. Responses to stimuli may vary from region to region due to aspects like culture, preferences and attitudes.

**Methods:**

We perform a spatial modeling of HIV and HSV-2 among women in Kenya, while relaxing these assumptions i.e. the linearity assumption by allowing the covariate age to have a non-linear effect on HIV and HSV-2 prevalence using the random walk model of order 2 and the stationarity assumption by allowing the rest of the covariates to vary spatially using the conditional autoregressive model. The women data used in this study were derived from the 2007 Kenya AIDS indicator survey where women aged 15–49 years were surveyed. A full Bayesian approach was used and the models were implemented in R-INLA software.

**Results:**

Age was found to have a non-linear relationship with both HIV and HSV-2 prevalence, and the spatially varying coefficient model provided a significantly better fit for HSV-2. Age-at first sex also had a greater effect on HSV-2 prevalence in the Coastal and some parts of North Eastern regions suggesting either early marriages or child prostitution. The effect of education on HIV prevalence among women was more in the North Eastern, Coastal, Southern and parts of Central region.

**Conclusions:**

The models introduced in this study enable relaxation of two limiting assumptions in disease mapping. The effects of the covariates on HIV and HSV-2 were found to vary spatially. The effect of education on HSV-2 status for example was lower in North Eastern and parts of the Rift region than most of the other parts of the country. Age was found to have a non-linear effect on HIV and HSV-2 prevalence, a linearity assumption would have led to wrong results and hence interpretations. The findings are relevant in that they can be used in informing tailor made strategies for tackling HIV and HSV-2 in different counties. The methodology used here may also be replicated in other studies with similar data.

**Electronic supplementary material:**

The online version of this article (doi:10.1186/s12889-016-3022-0) contains supplementary material, which is available to authorized users.

## Background

The World Health Organization (WHO) places at more than 1 million, the number of people who acquire sexually transmitted infections (STI) daily. By 2013 more than 530 million (about 7.5 %) had the virus that causes genital herpes or the herpes simplex virus type 2 (HSV-2) [1]. Out of these, it is estimated that about 123.7 million or 23 % resided in sub-Saharan Africa, among whom 63 % were women [2]. HSV-2 prevalence in the age group 15–49 in sub-Saharan Africa region ranges from 30 to 80 % among women and from 10 to 50 % among men [3]. There were about 35 million individuals living with HIV in sub-Saharan Africa by the end of 2013 with 2.1 million new infections [4]. HSV-2 is associated with a two to three-fold increased risk of HIV acquisition and an up to five-fold increased risk of HIV transmission per-sexual act, and may account for 40 to 60 % of new HIV infections in populations where HSV-2 has a high prevalence [2]. HIV and HSV-2 share common risk factors e.g. education level, place of residence, and age among others. Therefore understanding the spatial distribution, the dynamics and the underlying factors that propagate the spread of these diseases will help in ultimately winning the war against them. STIs can have serious consequences beyond the immediate impact of the infection itself, through mother-to-child transmission (MTCT) of infections and chronic diseases. Drug resistance is a major threat to reducing the impact of STIs worldwide [1]. The national HIV and HSV-2 prevalence rates in Kenya within the adult population (15–64 years) were estimated to be as high as 5.6 % and 7.1 % respectively [5], with a wide gender and geographical variation. The North Eastern region had HIV prevalence of as low as 2.1 % while regions around Lake Victoria and the Western region had prevalence ranging from between 13–25 % [6]. HIV and HSV-2 prevalence by age have a non-linear relationship assuming an inverted U shape [6, 7]. HIV prevalence increases with age until it plateaus at between ages 25–35, then starts decreasing with increasing age. HSV-2 prevalence increases with age up to between ages 35–45 then begins to decline with increasing age.

In the conventional generalized linear regression models applied to spatial data, many studies have assumed stationarity in that the same stimulus of a disease predictor provokes the same response in all parts of the study region [8–10]. This assumption is highly untenable for spatial processes. This may be as a result of sampling variation, intrinsically different relationships across space e.g. attitudes, cultures, preferences and model misspecification. It is therefore realistic to assume that the regression coefficients vary across space [11]. The issue of spatial non-stationarity can be addressed by allowing the relationships we are measuring to vary over space through the geographically weighted regression (GWR) model where the weights applied to observations in a series of locally weighted regression models across the study area are determined by a spatial kernel function [11], or the Bayesian spatially varying coefficients process (BSVCP), where spatially varying coefficients are modeled as a multivariate spatial process [12]. In the BSVCP model as discussed by Assuncao et al, the covariates are allowed to vary spatially by assigning its coefficients the Bayesian autoregressive (BAR), simultaneous autoregressive (SAR) or the conditional autoregressive (CAR) model [13]. Assuncao et al applied the BSVCP to model agricultural development in Brazil. The model showed significant regional differences in agricultural development [14]. Evidence of spatially varying parameters, even against strong prior belief on the absence of such variation, can be indicative of spatial differences of database collection procedures e.g. large differences on underreporting rates [13]. Several studies that use the linear predictor class of models including both the general and generalized linear models assume that all the covariates in the study have a linear relationship with the response variable. This linear relationship may not hold for all variables as in our case; age, which has a non-linear relationship with the response variable. Our objective is to perform a spatial modeling analysis while relaxing the stationarity and the linearity assumption by respectively employing the BSVCP and the random walk model of order 2 to model HIV and HSV-2 among women in Kenya.

## Methods

### Data

The data for this study was obtained from the Kenya AIDS Indicator Survey (KAIS) which was carried out by the Kenyan government with financial support from the United States President’s Emergency Plan for AIDS Relief (PEPFAR) and the United Nations (UN). The main aim of the survey was to obtain high quality data on the prevalence of HIV and Sexually Transmitted Infections (STI) among adults and to assess the knowledge of HIV and STIs in the population.

The sampling frame for KAIS was the National Sample Survey and Evaluation Programme IV (NASSEP IV). It consisted of 1800 clusters comprising 1260 rural and 540 urban clusters; of these, 294 rural and 141 urban clusters were sampled for KAIS. The overall design for KAIS 2007 was a stratified, two-stage cluster sampling design. The first stage involved selecting clusters from NASSEP IV, and the second stage involved the selection of household for KAIS with equal probability in the urban-rural strata within the districts. A sample of 415 clusters and 10,375 households were systematically selected for KAIS. A uniform sample of 25 households per cluster was selected using an equal probability systematic sampling method.

The survey was twofold: A household questionnaire was used to collect the characteristics of the living environment and an individual questionnaire to collect information on demographic characteristics and the knowledge of HIV and STIs on men and women aged 15–64 years. A representative sample of households and individuals was selected from eight provinces in the country. Each individual was asked for consent to provide a venous blood sample for HIV and HSV-2 testing. More information on survey methodologies used in collecting the data is found in the final KAIS, 2007 report [6]. This study uses the 2007 data even though a new round of KAIS, 2012 [5] has been done. The final release of this new data had not been made hence the data was not available for use. This study uses the women’s data from the KAIS, 2007 survey. Information from 4864 women, aged 15–64 years who had provided venous blood for HIV and HSV-2 testing and also had full covariate information was used in the analysis. In the data, age was captured as both categorical and continuous while all other covariates were categorical. Readers are directed to the KAIS, 2007 report [6] for more information. An initial exploratory data analysis was carried out using a univariate standard logistic regression model to determine the association of each single covariate with the outcome variable (HIV and HSV-2 status). These variables were categorized into four groups, namely: demographic, social, biological and behavioral [9].

From this initial analysis, education level, age at first sex, perceived risk, partners in the last 1 year, marital status, place of residence, STI status in the last 1 year and age of the respondent were found to be associated with HIV and HSV-2 infection.

### Statistical model

The covariates were tested for significance by fitting a univariate standard logistic model between each single covariate with the outcome variables (HIV and HSV-2 status). The association was considered significant at 5 % significance level. These are shown in Tables 1 and 2.

**Table 1:** Exploratory data analysis for HIV

Variable	*P*-Value	Unadjusted OR
Demographic characteristics		
Place of residence (Ref Rural)		
Urban	0.001	0.749 (0.635, 0.884)
Age (Ref 15–19)	0.000	
20–24	0.000	2.825 (1.982, 4.026)
25–29	0.000	3.055 (2.133, 4.375)
30–34	0.000	4.656 (3.276, 6.618)
35–39	0.000	3.682 (2.544, 5.328)
40–44	0.000	2.796 (1.869, 4.181)
45–49	0.000	2.783 (1.858, 4.169)
50–54	0.000	2.347 (1.490, 3.696)
55–59	0.294	1.352 (0.770, 2.375)
60–64	0.173	0.487 (0.173, 1.371)
Social Characteristics		
Wealth Quantile (ref poorest)	0.525	
Second	0.652	1.058 (0.827, 1.353)
Middle	0.392	0.896 (0.696, 1.153)
Fourth	0.564	1.074 (0.843, 1.369)
Richest	0.592	0.938 (0.741, 1.186)
Media access (Ref No)		
Yes	0.257	0.913 (0.781, 1.068)
Education level (Ref none)	0.000	
Primary	0.386	1.078 (0.910, 1.276)
Secondary	0.574	0.929 (0.720, 1.200)
Higher	0.000	0.451 (0.303,0 .671)
MaritalStatus (Ref Married, 1 partner)	0.000	
Married, +2 partners	0.001	1.536 (1.192, 1.980)
Divorced/separated	0.000	2.503 (1.960, 3.197)
Widowed	0.000	3.301 (2.645, 4.120)
Never married	0.000	0.647 (0.510,0 .820)
Perceived-Risk (Ref No risk)	0.000	
Small Risk	0.000	0.325 (0.231,0 .457)
Moderate Risk	0.000	0.447 (0.335, 0.597)
Great Risk	0.574	0.916 (0.676, 1.242)
Age-first-sex (Ref Never had sex)	0.000	
Under 11	0.000	8.524 (3.569, 20.358)
Between 12–14	0.000	10.162 (5.774, 17.885)
Between 15–17	0.000	8.636 (5.034, 14.817)
Over 18	0.000	4.870 (2.833, 8.371)
Biological characteristics		
Had STI (Ref Yes)		
No	0.000	0.406 (0.277, 0.597)
Ever given birth (Ref Yes)		
No	0.061	0.405 (0.316,0 .519)
Behavioral Characteristics		
Partners in last 1 year (Ref No partner)	0.000	
1 partner	0.034	1.021 (0.314,0.812)
2 partners	0.665	1.232 (0.771,3.433)
3 or more partners	0.999	2.455 (1.759,11.233)
Travel away (didn’t stay away)	0.029	
Stayed away 1–2 times	0.015	1.241 (1.042, 1.477)
Stayed away 3–5 times	0.006	1.362 (1.092, 1.698)
Stayed away 6–10 times	0.451	1.170 (0.778, 1.761)
Stayed away > 11 times	0.748	0.894 (0.451, 1.772)

**Table 2:** Exploratory data analysis for HSV-2

Variable	*P*-Value	Unadjusted OR
Demographic characteristics		
Place of residence (Ref Rural)		
Urban	0.000	0.823 (0.746,0 .907)
Age (Ref 15–19)	0.000	
20–24	0.000	2.745 (2.254, 3.343)
25–29	0.000	4.374 (3.591, 5.329)
30–34	0.000	6.794 (5.559, 8.303)
35–39	0.000	8.299 (6.739,10.220)
40–44	0.000	9.389 (7.538, 11.694)
45–49	0.000	8.641 (6.936, 10.765)
50–54	0.000	8.378 (6.592, 10.649)
55–59	0.000	8.661 (6.720, 11.162)
60–64	0.000	5.751 (4.279, 7.729)
Social Characteristics		
Wealth Quantile (ref poorest)	0.051	
Second	0.011	1.199 (1.042, 1.381)
Middle	0.466	1.053 (.916, 1.212)
Fourth	0.001	1.279 (1.113, 1.469)
Richest	0.569	1.039 (0.910, 1.186)
Media access (Ref No)		
Yes	0.821	1.010 (0.924, 1.104)
Education level (Ref none)	0.000	
Primary	0.000	0.814 (0.738, 0.898)
Secondary	0.000	0.704 (0.610,0 .813)
Higher	0.000	0.457 (0.381, 0.548)
Marital Status (Ref Married, 1 partner)	0.000	
Married, +2 partners	0.000	2.381 (2.042, 2.778)
Divorced/separated	0.000	1.904 (1.607, 2.256)
Widowed	0.000	3.238 (2.719, 3.857)
Never married	0.000	0.292 (0.257,0 .333)
Perceived-Risk (Ref No risk)	0.000	
Small Risk	0.000	0.452 (0.371,0 .551)
Moderate Risk	0.000	0.581 (0.483, 0.699)
Great Risk	0.675	0.957 (0.778, 1.177)
Age-first-sex (Ref Never had sex)	0.000	
Under 11	0.000	12.572 (7.554, 20.922)
Between 12–14	0.000	18.384 (13.685, 24.697)
Between 15–17	0.000	15.053 (11.477, 19.743)
Over 18	0.000	9.797 (7.487, 12.818)
Biological characteristics		
Had STI (Ref Yes)		
No	0.000	0.556 (0.407,0 .760)
Ever given birth (Ref Yes)		
No	0.052	0.187 (0.163, 0.215)
Behavioral Characteristics		
Partners in last 1 year (Ref No partner)	0.009	
1 partner	0.802	0.990 (0.873,1.276)
2 partners	0.831	1.108 (1.925, 6.294)
3 or more partners	0.938	0.535 (0.699,1.434)
Travel away (didn’t stay away)	0.000	
Stayed away 1–2 times	0.000	1.251 (1.133, 1.380)
Stayed away 3–5 times	0.000	1.468 (1.289, 1.672)
Stayed away 6–10 times	0.017	1.324 (1.052, 1.665)
Stayed away > 11 times	0.198	1.258 (0.887, 1.786)

Let *y*_*ijk*_ be the disease *k* status (0/1), *k* = 1 for HIV and *k* = 2 for HSV-2, for individual *j* in county *i*: *i* = 1, 2, …, 46. *y*_*ij*1_ = 1 if individual *j* in county *i* is HIV positive and zero otherwise and *y*_*ij*2_ = 1 if individual *j* in county *i* is HSV-2 positive and zero otherwise. This study assumes the dependent variable *y*_*ij*1_ *and y*_*ij*2_ are univariate Bernoulli distributed, i.e. *y*_*ij*1_|*p*_*ij*1_ ~ *Bernoulli*(*p*_*ij*1_) and *y*_*ij*2_|*p*_*ij*2_ ~ *Bernoulli*(*p*_*ij*2_).

The *p* continuous independent variables are contained in the vector ***X***_***ijk***_ = (*x*_*ij*1_, *x*_*ij*2_, …, *x*_*ijp*_)^'^ while ***W***_***ijk***_ = (*w*_*ij*1_, *w*_*ij*2_, …, *w*_*ijr*_)^'^ contains *r* categorical independent random variables with the first component accounting for intercept. In this study, *p* = 1 (*age*) and *r* = 8_._

The unknown mean response namely *E*(*y*_*ijk*_) = *p*_*ijk*_ relates to the independent variable as follows:

$$ \begin{array}{l}h\left({p}_{ij1}\right)={\boldsymbol{X}}^{\boldsymbol{T}}{\boldsymbol{\beta}}_{\boldsymbol{1}}+{\boldsymbol{W}}^{\boldsymbol{T}}{\boldsymbol{\gamma}}_{\boldsymbol{1}},\ \mathrm{f}\mathrm{o}\mathrm{r}\ \mathrm{H}\mathrm{I}\mathrm{V}\ \mathrm{and}\\ {}h\left({p}_{ij2}\right)={\boldsymbol{X}}^{\boldsymbol{T}}{\boldsymbol{\beta}}_{\boldsymbol{2}}+{\boldsymbol{W}}^{\boldsymbol{T}}{\boldsymbol{\gamma}}_{\boldsymbol{2}}\ \mathrm{f}\mathrm{o}\mathrm{r}\ \mathrm{H}\mathrm{S}\mathrm{V}\hbox{-} 2.\end{array} $$

Where *h*(.) is a logit link function, **β** is a *p* dimensional vector of regression coefficients for the continuous independent variables, and **γ** is a *r* dimensional vector of regression coefficients for the categorical independent variables. A random walk model of order 2 (RW2) and a convolution model were employed in order to cater for both the non-linear effects of the continuous covariates and the spatial autocorrelation in the data.

The RW2 model approach relaxed the highly restrictive linear predictor by a more flexible semi-parametric predictor, defined as:

$$ \begin{array}{l}h\left({p}_{ij1}\right)={\displaystyle \sum_{t=1}^p{f}_t\left({x}_{ijt}\right)+{f}_{spat}\left({s}_{i1}\right)}+{\boldsymbol{W}}^{\boldsymbol{T}}{\boldsymbol{\gamma}}_{\boldsymbol{1}}\ \mathrm{f}\mathrm{o}\mathrm{r}\ \mathrm{H}\mathrm{I}\mathrm{V}\ \mathrm{and}\\ {}h\left({p}_{ij2}\right)={\displaystyle \sum_{t=1}^p{f}_t\left({x}_{ijt}\right)+{f}_{spat}\left({s}_{i2}\right)}+{\boldsymbol{W}}^{\boldsymbol{T}}{\boldsymbol{\gamma}}_{\boldsymbol{2}}\ \mathrm{f}\mathrm{o}\mathrm{r}\ \mathrm{H}\mathrm{S}\mathrm{V}\hbox{-} 2\end{array} $$

The function *f*_*t*_(.) is a non-linear twice differentiable smooth function for the continuous covariate and *f*_*spat*_(*s*_*ik*_) is a factor that caters for the spatial effects of each county. This study utilized the convoluted spatial structure which assumes that the spatial effect can be decomposed into two components: spatially structured and spatially unstructured i.e. *f*_*spat*_(*s*_*ik*_) = *f*_*str*_(*s*_*ik*_) + *f*_*unstr*_(*s*_*ik*_) , *k* = 1, 2 [9, 15]. The spatially unstructured random effects cover the unobserved covariates that are inherent within the counties or the correlation within the counties e.g. common cultural practices, climate, cultures etc. while the spatially structured random effect accounts for any unobserved covariates which vary spatially among counties. This is called spatial autocorrelation and it is technically defined as the dependence due to geographical proximity. Thus the final model is expressed as:

$$ h\left({p}_{ijk}\right)={\displaystyle \sum_{t=1}^p{f}_t\left({x}_{ijt}\right)+{f}_{str}\left({s}_{ik}\right)+{f}_{unstr}\left({s}_{ik}\right)}+{\boldsymbol{W}}^{\boldsymbol{T}}{\boldsymbol{\gamma}}_{\boldsymbol{k}}, $$

$$ with\;k=1\;for\;HIV\; and\;k=2\;for\;HSV-2 $$

### Parameter estimation

This study used a full Bayesian estimation approach where parameters were assigned prior distributions as will be discussed in the priors’ specification section.

### Non-linear effects

Several studies have discussed extensively the methods for estimating the smooth function *f*_*t*_(.) [16–18]. The penalized regression splines model proposed by Eliers and Marx [18] for example is commonly used. Here, the assumption is that the effect of the continuous covariates can be approximated using the polynomial spline. They assumed that the smooth function *f*_*t*_(.) can be estimated by a spline of degree *l* with *K* equally spaced knots; *x*_*p*,min_ = *ψ*_*p*1_ < *ψ*_*p*2_ ⋯ < *ψ*_*pk* − 1_ < *ψ*_*pK*_ = *x*_*p*,max_. Many studies have explored the relationships between the Gaussian Markov Random Fields (GMRF) and smoothing splines [19–21].In this study we used the random walk model for estimating the smooth function *f*_*t*_(.). This is briefly discussed in Appendix 1.

### Spatially varying coefficients

As stated before, many studies have been done with the assumption that the relationship between the explanatory variable and the response variables in a regression model are constant across the study region [8–10]. This assumption is unrealistic for spatial processes as factors such as sampling variation, different relationships across space e.g. attitudes, preferences, culture etc. contribute to a different response to the same stimuli as one moves across space. Two competing spatially varying models are the GWR and the BSVCP. The GWR addresses this by estimating the coefficients **β**^'^**s** by the weighted least squares method, where more emphasis in terms of weights are placed on the observations which are close to location *i*, since it is assumed that the observations close to *i* exert more influence on the parameter estimates at location *i* than those farther away [11]. The weighting schemes can be fixed or adaptive. In the fixed scheme, observations that are within some distance *d* are given the weight of 1 while those farther away beyond some distance *d* from location *i* are given a weight of zero, while in the adaptive scheme, weights inside some radius *d* are made to decrease monotonically to zero as the radius increases. In this study we used the BSVCP (Appendix 2) model to relax the stationarity assumption, the covariates are allowed to vary spatially by assigning its coefficients the conditional autoregressive (CAR) model [13].

### Priors for the spatial components

The prior for the structured random effects was defined to follow the CAR model while for the unstructured random effects, the independently and identically distributed normal distribution.

### Posterior distribution

This is the distribution of the parameters after observing the data. The posterior distribution is obtained by updating the prior distribution with the observed data. Since our study is fully Bayesian, inference is made by sampling from this posterior distribution. Markov Chain Monte Carlo (MCMC) is the most common approach to do inference for latent Gaussian models however this method is slow and performs poorly when applied to such models [22]. The Integrated Nested Laplace (INLA) criterion is a relatively new technique developed to circumvent these shortfalls [22]. The posterior distribution for the latent Gaussian model is:

$$ \pi \left(\boldsymbol{x},\boldsymbol{\theta} \left|\boldsymbol{y}\right.\right)\;\alpha\;\pi \left(\boldsymbol{\theta} \right)\pi \left(\boldsymbol{x}\left|\boldsymbol{\theta} \right.\right)\prod_{i\in I}\pi \left({y}_i\left|{x}_i,\boldsymbol{\theta} \right.\right) $$

$$ \alpha\;\pi \left(\boldsymbol{\theta} \right){\left|\boldsymbol{Q}\left(\boldsymbol{\theta} \right)\right|}^{\frac{n}{2}} \exp \left(-\frac{1}{2}{x}^TQ\left(\boldsymbol{\theta} \right)x+{\displaystyle \sum_{i\in I} \log \pi \left({y}_i\left|{x}_i,\boldsymbol{\theta} \right.\right)}\right). $$

Where ***x*** is the class of latent fields, ***θ*** is the set of hyper parameters and ***y*** is the data. In the INLA approach, the posterior marginals of interest are:

$$ \pi \left({x}_i\left|\boldsymbol{y}\right.\right)={\displaystyle \int \pi \left({x}_i\left|\boldsymbol{\theta}, \boldsymbol{y}\right.\right)}\;\pi \left(\boldsymbol{\theta} \left|\boldsymbol{y}\right.\right)d\boldsymbol{\theta}\ \mathrm{and}\ \pi \left({\theta}_j\left|\boldsymbol{y}\right.\right)={\displaystyle \int \pi \left(\boldsymbol{\theta} \left|\boldsymbol{y}\right.\right)d{\boldsymbol{\theta}}_{\mathit{\hbox{-}}j}}, $$

and these are used to construct the nested approximations:

$$ \tilde{\pi}\left({x}_i\left|\boldsymbol{y}\right.\right)={\displaystyle \int \tilde{\pi}\left({x}_i\left|\boldsymbol{\theta}, \boldsymbol{y}\right.\right)}\;\tilde{\pi}\left(\boldsymbol{\theta} \left|\boldsymbol{y}\right.\right)d\boldsymbol{\theta} \kern0.5em \mathrm{and}\ \tilde{\pi}\left({\theta}_j\left|\boldsymbol{y}\right.\right)={\displaystyle \int \tilde{\pi}\left(\boldsymbol{\theta} \left|\boldsymbol{y}\right.\right)d{\boldsymbol{\theta}}_{\mathit{\hbox{-}}j}}. $$

The analyses in this study were carried out using the R software with the INLA package. The codes used for this analysis can be found in Additional file 1.

### Model diagnostics

The models were compared using the deviance information criterion (DIC) suggested by Spiegelhalter et al. [23]. The best fitting model is one with the smallest DIC. The DIC value is obtained as: $DIC=\overline{D}\left(\theta \right)+pD$, where $\overline{D}$ is the posterior mean of the deviance that measures the goodness of fit while *pD* gives the effective number of parameters in the model which penalizes for complexity of the model. In DIC, low values of $\overline{D}$ indicate a better fit while small values of *pD* indicate model parsimony. One challenge with the DIC is, how big the difference in DIC values of two competing models needs to be in order to declare one model as being better than the other is not well defined. Studies have declared that a difference in DIC of 3 between two models cannot be distinguished while for a difference of between 3 and 7 the two models can be weakly differentiated [23].

### Application/Data analysis

The following sets of models were investigated in order to understand the effect of the observed covariates and unobserved effects on the distribution of HIV and HSV-2 in Kenya among the female population.

**Model 1:** This is a model of fixed categorical covariates which are assumed to have linear effects on the response variable namely, education level, age at first sex, perceived risk, partners in the last 1 year, marital status, place of residence, STI status in the past 1 year, number of times one had stayed away from home in the past 1 year and one continuous covariate, age, modeled with a non-linear smooth function: the RW2 model. Model 1 does not take into account the spatially structured and the spatially unstructured random effects and the two diseases are modeled independently.

**Model 2:** This is an additive model that assumes linear effects of the categorical covariates listed in model 1 above, non-linear effect of the continuous covariate age and spatially unstructured random effect which caters for the unobserved covariates that are inherent within the counties specified by the identically and independently distributed (iid) normal distribution.

**Model 3:** This model explores the effect of the linear covariates listed in model 1 above, non-linear covariate age and spatially structured random effect which accounts for any unobserved covariates which vary spatially among counties, specified by the CAR model.

**Model 4:** Examines the effects of the nonlinear effects of age, linear effects of the categorical covariates and a convolution of spatially structured and spatially unstructured random effect, specified by the CAR model and the iid normal distribution respectively.

**Models 5–8** are similar to models 1–4 respectively, the only difference is that the regression coefficients ***γ*** in these models are assumed to vary spatially and are assigned CAR priors.

$$ \begin{array}{l} Mode{l}_1: logit\left({\rho}_{ij1}\right)={\beta}_{01}+f(age)+{\boldsymbol{w}}^{\boldsymbol{T}}\boldsymbol{\gamma}\;for\;HIV\\ {}\kern2.04em  logit\left({\rho}_{ij2}\right)={\beta}_{02}+f(age)+{\boldsymbol{w}}^{\boldsymbol{T}}\boldsymbol{\gamma}\;for\;HSV-2\end{array} $$

$$ \begin{array}{l} Mode{l}_2: logit\left({\rho}_{ij1}\right)={\beta}_{01}+f(age)+{\boldsymbol{w}}^{\boldsymbol{T}}\boldsymbol{\gamma} +{f}_{unstr}\left({s}_{i1}\right)\;for\;HIV\\ {}\kern2.28em  logit\left({\rho}_{ij2}\right)={\beta}_{02}+f(age)+{\boldsymbol{w}}^{\boldsymbol{T}}\boldsymbol{\gamma} +{f}_{unstr}\left({s}_{i2}\right)\;for\;HSV-2\end{array} $$

$$ \begin{array}{l} Mode{l}_3: logit\left({\rho}_{ij1}\right)={\beta}_{01}+f(age)+{\boldsymbol{w}}^{\boldsymbol{T}}\boldsymbol{\gamma} +{f}_{str}\left({s}_{i1}\right)\;for\;HIV\\ {}\kern2.28em  logit\left({\rho}_{ij2}\right)={\beta}_{02}+f(age)+{\boldsymbol{w}}^{\boldsymbol{T}}\boldsymbol{\gamma} +{f}_{str}\left({s}_{i2}\right)\;for\;HSV-2\end{array} $$

$$ \begin{array}{l} Mode{l}_4: logit\left({\rho}_{ij1}\right)={\beta}_{01}+f(age)+{\boldsymbol{w}}^{\boldsymbol{\prime}}\boldsymbol{\gamma} +{f}_{unstr}\left({s}_{i1}\right)+{f}_{str}\left({s}_{i1}\right)\;for\;HIV\\ {}\kern2.28em  logit\left({\rho}_{ij2}\right)={\beta}_{02}+f(age)+{\boldsymbol{w}}^{\boldsymbol{\prime}}\boldsymbol{\gamma} +{f}_{unstr}\left({s}_{i2}\right)+{f}_{str}\left({s}_{i2}\right)\;for\;HSV-2\end{array} $$

$$ \begin{array}{l} Mode{l}_5: logit\left({\rho}_{ij1}\right)={\beta}_{01}+f(age)+{\boldsymbol{w}}^{\boldsymbol{T}}\boldsymbol{\gamma}\;for\;HIV\\ {}\kern2.04em  logit\left({\rho}_{ij2}\right)={\beta}_{02}+f(age)+{\boldsymbol{w}}^{\boldsymbol{T}}\boldsymbol{\gamma}\;for\;HSV-2\end{array} $$

$$ \begin{array}{l} Mode{l}_6: logit\left({\rho}_{ij1}\right)={\beta}_{01}+f(age)+{\boldsymbol{w}}^{\boldsymbol{T}}\boldsymbol{\gamma} +{f}_{unstr}\left({s}_{i1}\right)\;for\;HIV\\ {}\kern2.28em  logit\left({\rho}_{ij2}\right)={\beta}_{02}+f(age)+{\boldsymbol{w}}^{\boldsymbol{T}}\boldsymbol{\gamma} +{f}_{unstr}\left({s}_{i2}\right)\;for\;HSV-2\end{array} $$

$$ \begin{array}{l} Mode{l}_7: logit\left({\rho}_{ij1}\right)={\beta}_{01}+f(age)+{\boldsymbol{w}}^{\boldsymbol{T}}\boldsymbol{\gamma} +{f}_{str}\left({s}_{i1}\right)\;for\;HIV\\ {}\kern2.28em  logit\left({\rho}_{ij2}\right)={\beta}_{02}+f(age)+{\boldsymbol{w}}^{\boldsymbol{T}}\boldsymbol{\gamma} +{f}_{str}\left({s}_{i2}\right)\;for\;HSV-2\end{array} $$

$$ \begin{array}{l} Mode{l}_8: logit\left({\rho}_{ij1}\right)={\beta}_{01}+f(age)+{\boldsymbol{w}}^{\boldsymbol{\prime}}\boldsymbol{\gamma} +{f}_{unstr}\left({s}_{i1}\right)+{f}_{str}\left({s}_{i1}\right)\;for\;HIV\\ {}\kern2.28em  logit\left({\rho}_{ij2}\right)={\beta}_{02}+f(age)+{\boldsymbol{w}}^{\boldsymbol{\prime}}\boldsymbol{\gamma} +{f}_{unstr}\left({s}_{i2}\right)+{f}_{str}\left({s}_{i2}\right)\;for\;HSV-2\end{array} $$

### Ethical statement

Ethical clearance was granted by the institutional review board of the Kenya Medical Research Institute (KEMRI) and the US Centers for Disease Control and Prevention. No ethical clearance was required from the University of Kwazulu-Natal or any other institution save for the aforementioned. The consent procedure, stated below, was approved by KEMRI and the US centers for Disease Control and Prevention.

Participants provided separate informed oral consent for interviews, blood draws and blood storage and the interviewer signed the consent form to indicate whether or not consent was given for each part. An oral informed consent was given for participants in the age of 18–64 while for minors, in the age group 15–17, oral informed consent was obtained from a parent/guardian or other adult responsible for the youth’s health and welfare before the youth was asked for his/her consent. Only after the parent or guardian had agreed, was the consent of the adolescent sought.

Investigators in the study got a waiver of documentation of informed consent for all participants due to the fact that the research presented very minimal risk of harm to the individuals. The waiver did not adversely affect the rights and welfare of the participants, and the survey involved no procedures for which written consent is normally required outside the research context in Kenya.

## Results

### Model assessment and comparison

Table 3 shows the DICs for the four separately fitted models for HIV and HSV-2. These four models were assumed to have stationary coefficients. Table 4 shows the DICs for the four separate models with spatially varying coefficients. The model with the smallest DIC provides the best fit. Studies have however reported that two models with a difference of 3 or less in DIC are indistinguishable, while a difference of between 3 and 7 suggests that the two models are weakly distinguishable [23]. From the tables, all the spatially varying models have a lower DIC as compared with their corresponding stationary models. For HIV, Spatially varying coefficient models 6, 7, 8 are not significantly different form each other and from the corresponding stationary model counterparts as the difference in DIC is less than 3. This suggests that the covariates for HIV do not vary significantly across space. For HSV-2, the spatially varying models are significantly better than the stationary models since they have significantly lower DICs. This suggests that the covariates provoke different responses across space for HSV-2. Spatially varying model 8 provided the best fit for HSV-2.

**Table 3:** Stationary model

	Model 1		Model 2		Model 3		Model 4	
	HIV	HSV-2	HIV	HSV-2	HIV	HSV-2	HIV	HSV-2
*pD*	12.83	13.71	38.50	51.28	38.84	51.17	38.47	51.28
$\overline{D}\left(\theta \right)$	2509.47	6202.83	2366.25	5827.92	2367.05	5827.87	2366.24	5827.90
Total DIC	2522.30	6216.54	2404.75	5879.20	2405.89	5879.04	2404.71	5879.18

**Table 4:** Spatially varying coefficients

	Model 5		Model 6		Model 7		Model 8	
	HIV	HSV-2	HIV	HSV-2	HIV	HSV-2	HIV	HSV-2
*pD*	32.43	61.70	38.68	69.58	39.05	68.57	38.58	69.34
$\overline{D}\left(\theta \right)$	2430.02	5932.32	2365.98	5773.91	2365.77	5779.05	2365.80	5773.84
Total DIC	2462.45	5994.02	2404.66	5843.49	2404.82	5847.62	2404.38	5843.17

We therefore present and discuss the results based on model 8 for both HIV and HSV-2, which allows the covariates to vary spatially by the CAR model and also captures the structured and the unstructured random effects.

### Spatially varying effects

**Fig. 1:**
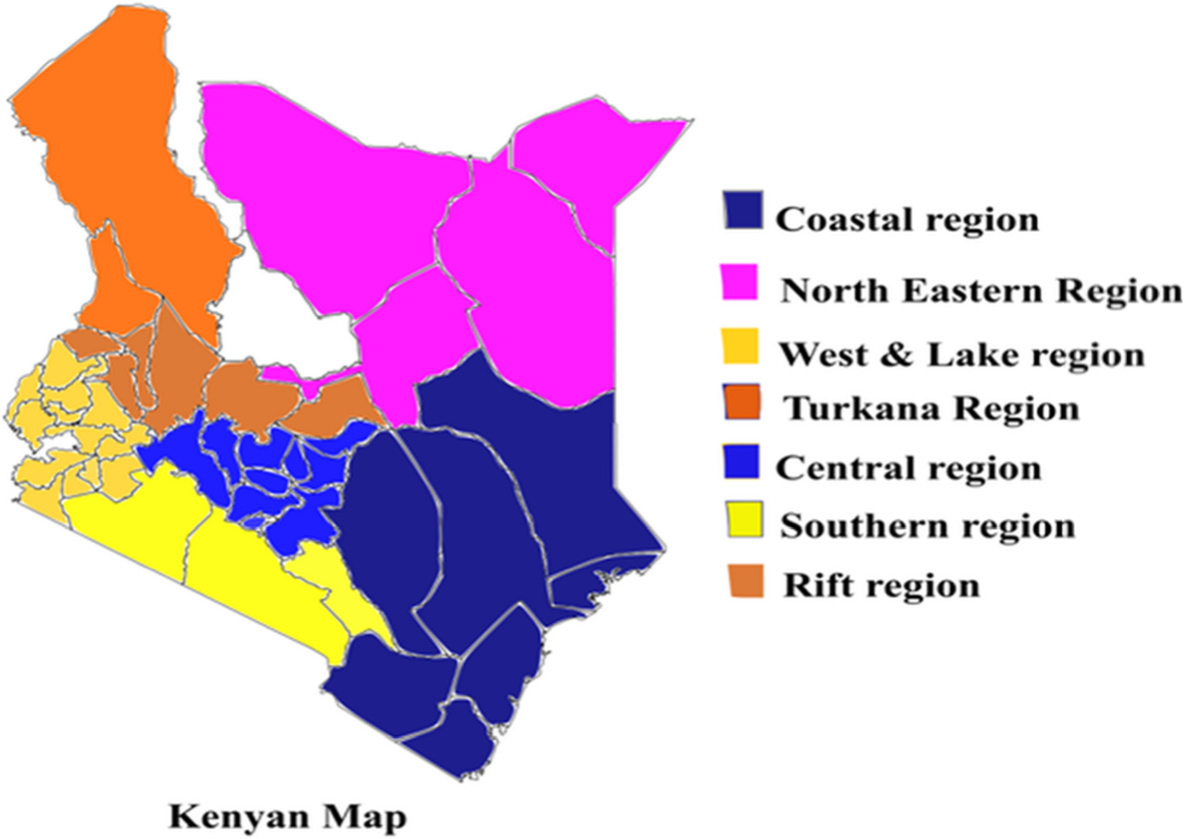
Map of Kenya

The DIC values indicate that the SVC models are better than the stationary ones, especially for the HSV-2 model. The choropleth maps show the varying effects of each covariate across space. Figure 1 shows the map of Kenya. Kenya is positioned on the equator on Africa’s East Coast. The administration units in Kenya were provinces before changing to counties after the 2010 promulgation of the constitution. There are 47 counties in Kenya but this study discusses results from 46 counties as the KAIS 2007 was not conducted in Samburu County due to insecurity.

### HIV

**Fig. 2:**
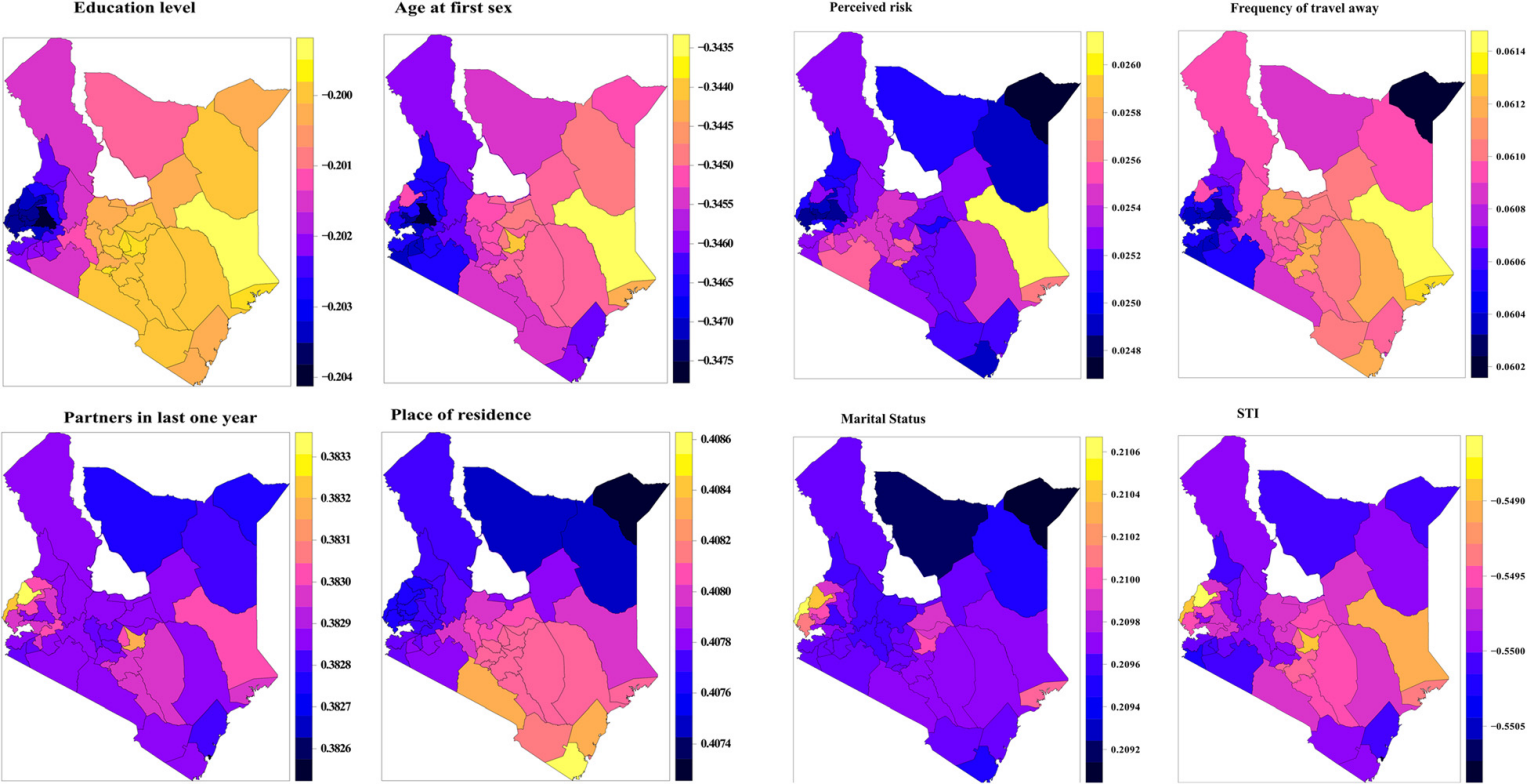
Spatially varying effects of covariates on HIV status

Though the SVC model for HIV was not significantly different from its stationary counterpart, the choropleth maps suggest that the effects of some of the covariates vary across space. The effect of education on HIV prevalence among women was more in the North Eastern, Coastal, Southern regions and parts of Central region indicated by the yellow to orange shading in the choropleth map in Fig. 2. Age at first sex also had a greater effect in those parts where education had greater effects as compared with the other parts of the country suggesting a correlation between education and age at first sex. The effect of number of partners had in the last 1 year was almost the same across the country except for some parts of West, Lake and Central region, where the effect was greater indicated by yellow/orange shading on the choropleth map in Fig. 2. The effect of frequency of travel away was also evident in the North Eastern, Coastal and Southern regions and parts of Central region while that of marital status was dominant in the Lake region.

### HSV-2

**Fig. 3:**
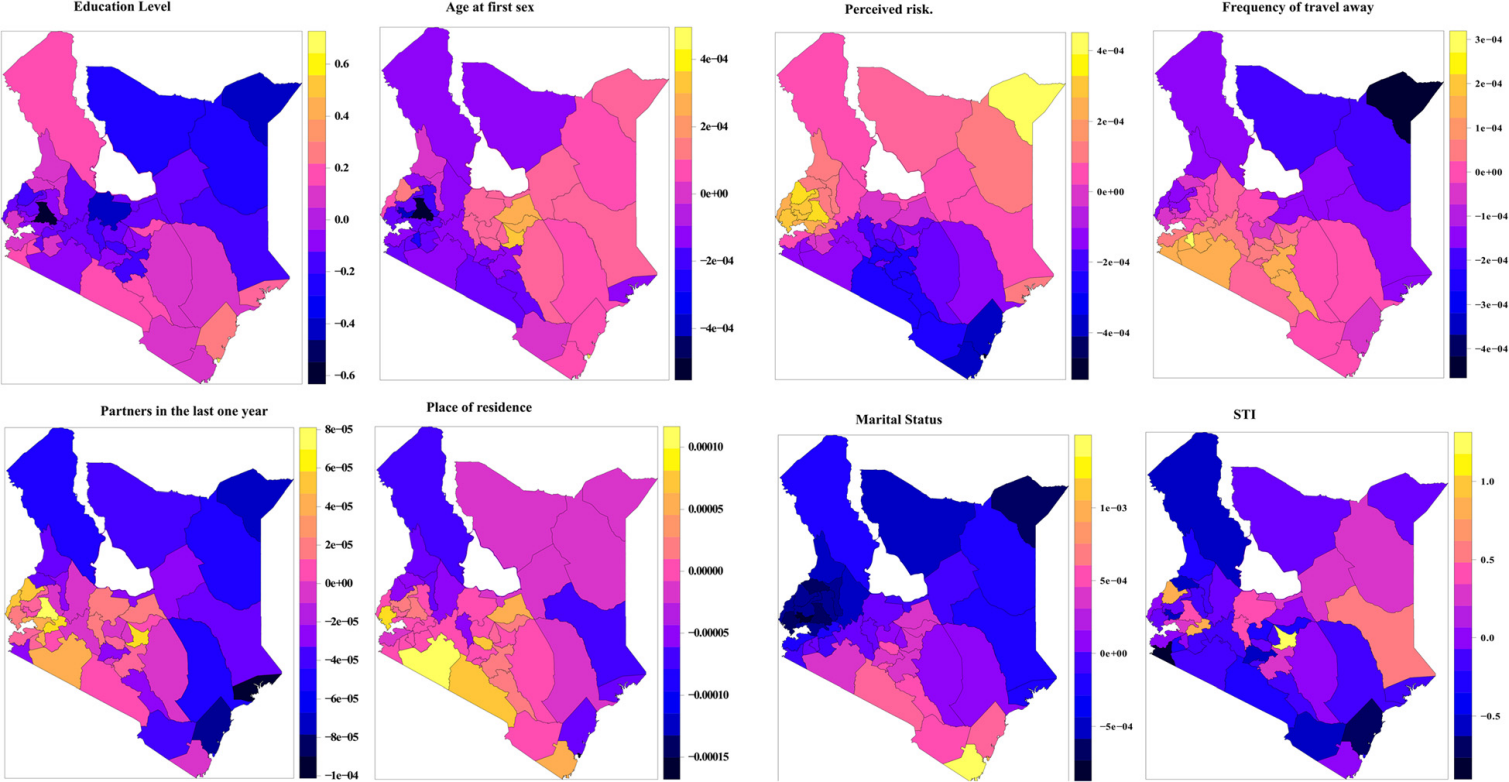
Spatially varying effects of covariates on HSV-2 status

The effect of education on HSV-2 status was lower in North Eastern and parts of Rift region than most of the other parts of the country shown by the blue shading on the map in Fig. 3. Age at first sex also had a greater bearing in the Costal and some parts of North Eastern, parts of Rift and West and Lake (pink/yellow shading) suggesting either early marriages or child prostitution. The highest rates of arranged marriages among adolescent girls in Kenya are found in Northeastern (73 %), Rift Valley (22 %), and Coast (21 %) provinces [24]. A study by the University of Chicago in Kenya and Zambia found that among 15-to-19 year old girls who are sexually active, being married increased their chance of HIV and other STIs by more than 75 %. This is due to the fact that most of these young marrieds were more likely to be in a polygamous union [25]. Partners had in the last 1 year had more effect on HSV-2 status in the West and Lake regions and some parts of the Central and Southern regions depicted by yellow shading on Fig. 3, while the number of partners had in the last 1 year had less effect in the regions with blue shading. The effect of place of residence also varied spatially. The effects were higher in the West and Lake, Southern and parts of Central and Coastal and Rift regions depicted by yellow shading on Fig. 3.

**Fig. 4:**
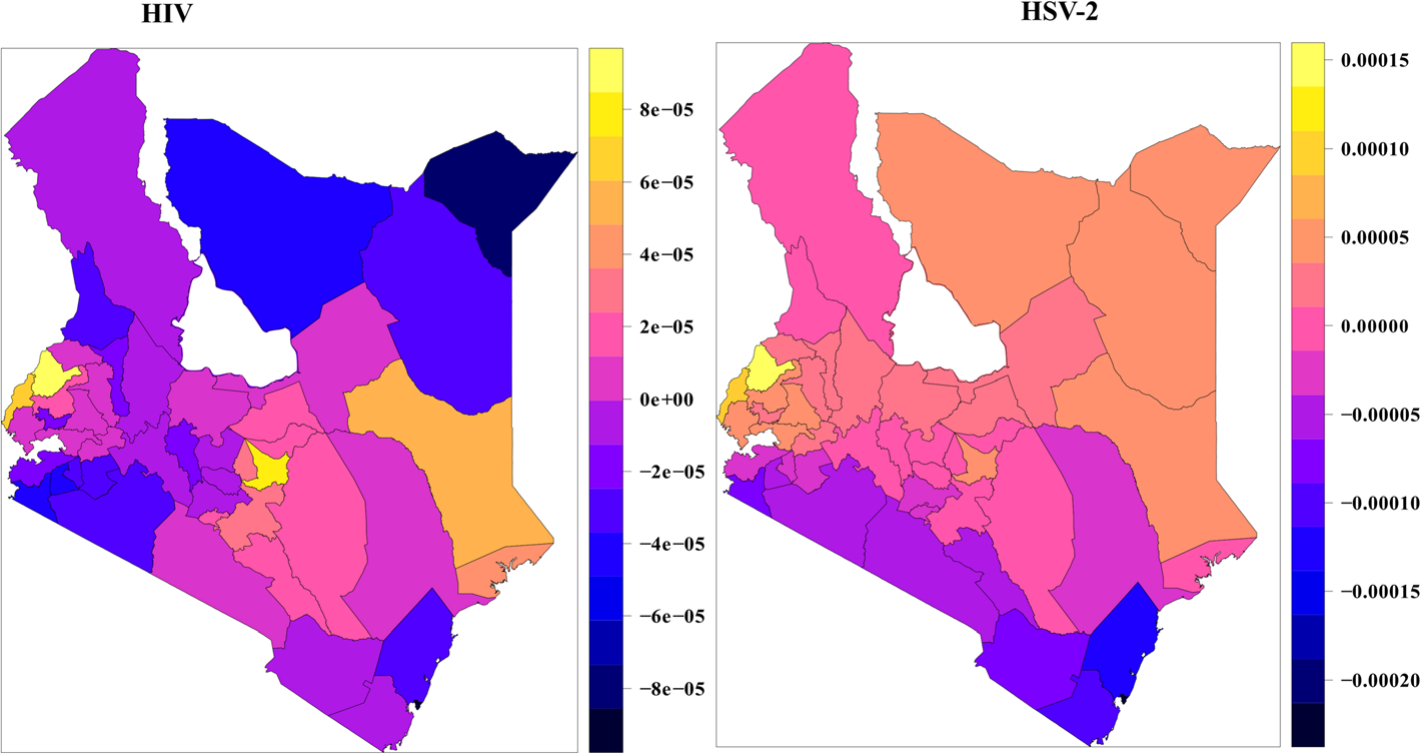
Spatial effects of HIV and HSV-2

The spatial effects based on model 4 indicate that HIV prevalence varies spatially with areas in the Central, West and Lake regions recording the highest prevalence. HIV prevalence is lowest in the North Eastern region (shown by blue shadding on Fig. 4) with some significant prevalence in some parts of the Coastal region. On the other hand, HSV-2 prevalence is also highest in the West and Lake regions, but also generally high across the country as shown in the yellow/orange shadding on the choropleth map in Fig. 4. Most regions with high HSV-2 prevalence had aslo a high HIV prevalence. Identifying the effects of individual covariates on each area can go a long way in informing strategies to deal with HIV and HSV-2 prevalence.

### The non-linear effect of age

**Fig. 5:**
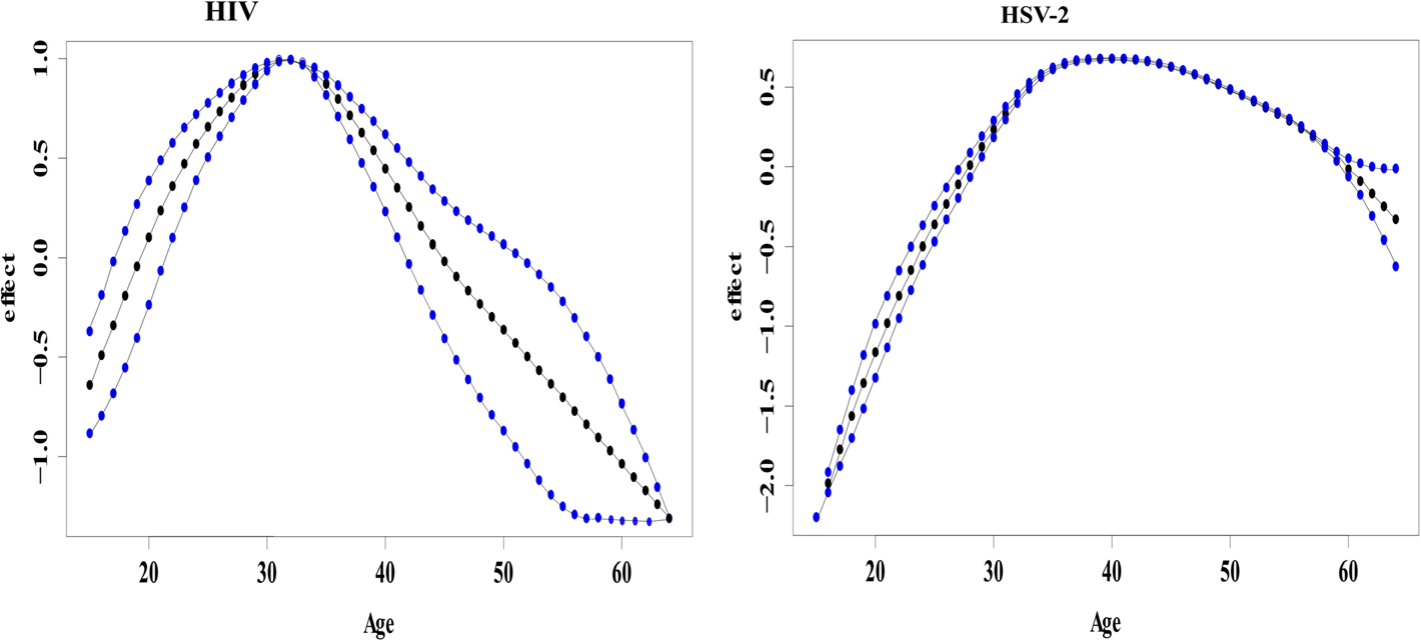
Non-linear effect of age on HIV and HSV-2

Figure 5 shows the nonlinear association between age of an individual and HIV infection and age of an individual and HSV-2 infection. The figures give the posterior mean of the smooth function and their corresponding 95 % CI. From the figures it is evident that there is a nonlinear relationship between age and HIV and HSV-2 infection. An assumption of linear relationship would have led to misleading results and subsequently wrong interpretations. The chance of HIV infection increases with age up to an optimum age of about 30 years then starts declining with increase in age. For HSV-2, the likelihood of infection increases with age up to an optimum age of about 40 years then starts to decline thereafter with increasing age. The results depict that the prevalence of HIV peaks earlier in age than HSV-2.

## Discussion

This study found that the effect of the covariates on HIV and HSV-2 prevalence varied spatially, although the spatially varying HIV model was not significantly different from the stationary one. This could be due to bias introduced by deletion of cases. A stationarity assumption would therefore have masked these varying effects. The major strength of the spatially varying model is that it is able to unmask the effect of each covariate on HIV and HSV-2 prevalence in each region. Age at first sex had greatest effect on HSV-2 prevalence in the Central and parts of Rift region and more effect on HIV prevalence in the Coastal, North Eastern and Central regions. This may suggest either early marriages,child prostitution or teenage sex. Intervention strategies geared towards delaying the age at first sex, stoping childhood prostitution or early marriages can be put in place in these regions. Partners had in the last 1 year had more effect on HSV-2 status in the West and Lake regions and some parts of the Central region. Residents in these regions can be educated on faithfulness, use of protection and/or absteinance. Place of residence had more effect on HSV-2 prevalence in the Southern, parts of Central, West, Lake and Coastal regions. Various studies have documented that education level is inversely related to HIV and HSV-2 infection [26, 27]. Education level provoked more response in HIV prevalence in the North Eastern, Coastal, Southern and parts of Central region. In the Coastal region where tourism is rife, vices such as child prostitution and drug abuse can greatly contribute to the prevalence of HIV and HSV-2. Education can not only detract an individual from activities that can lead to acquisition of HIV and/or HSV-2, but also make them aware of the safe practices. The effects of frequency of travel away on HIV prevalence was dominant in Coastal, Central and Rift regions, with some parts of North Eastern region having a near zero effect while for HSV-2 prevalence, the effect was dominant in the West and Lake regions and some parts of Central and Rift region. This shows that frequency of travel away has different effects across the regions suggesting that women in the Coastal, Central and Rift regions travel away from their homes/regions more than women from the rest of the country. Frequency of travel away also has different effects on HIV and HSV-2. Since its effect on HSV-2 is dominant in West and Lake region, this could mean that the regions visited by these women have high HSV-2 prevalence and the same applies for HIV. The 2011-12 Tanzanian HIV/AIDS and malaria indicator survey found that women who traveled away from home five or more times in a year were twice likely to be infected with HIV(STIs) compared to women who did not travel [28]. This could be due to the fact that these women are more likely to engage in risky sexual behaviours when they are away from home. The effect of marital status on HIV prevalence was dominant in the West and the Lake region. This could be attributed to traditional practices such as wife inheritance which is rife in these regions. Wife inheritance is a widespread cultural practice in sub-Saharan Africa that increases the risk of HIV acquisition and transmission [29, 30]

Age was found to have a non-linear effect on both HIV and HSV-2. i.e. an inverted “U” shape. The likelihood of HIV infection among women increases with age up to about age 30 then reduces thereafter with increasing age. On the other hand the likelihood of HSV-2 infection increases with age up to about age 40 and then starts declining with age. These findings were consistent with other studies [31]. Spatial effects in the model account for unobserved variables that represent those variables that vary spatially. Identifying high prevalence areas and the relationship between HIV and HSV-2 can provide more insight that can be useful in coming up with campaigns and prevention strategies for specific regions. There was evidence of spatial variation of HIV and HSV-2 infection among counties. HIV prevalence was lowest in the North Eastern region with some significantly high prevalence in some parts of the Coastal, Central, Western and lake regions. HSV-2 prevelance was highest in the West and Lake regions, but generally high across the country. Identifying the effects of individual covariates on each region will help in informing region specific strategies to deal with HIV and HSV-2 prevalence.

The spatially varying coefficient model has a huge epidemiological implication. With limited resources such as funds, time and personnel, intervention strategies may be tailor made for specific regions instead of rolling out blanket intervention strategies. More emphasis for example can be put in delaying the age at first sex in those regions where the effect of age at first sex on HIV and HSV-2 was great etc. Areas where individuals engage in sexual activities with multiple partners can for example be targeted with intervention strategies tailored to either help these individuals stick to one partner or educate them on the use of protection rather than addressing issues that do not contribute much to the prevalence of HIV and HSV-2 in that particular area thereby wasting valuable resources.

## Conclusion

This study used a full Bayesian approach to relax the stationarity assumption of the coefficients using the conditional autoregressive model [12]. The non-linear effects of age were modeled using the random walk model of order 2 [32], while the spatial effects and the spatially unstructured random effects in the model were modeled using a Gaussian Markov Random Field (GMRF) and a zero mean Gaussian process respectively. We determined that the effects of the covariates on HIV and HSV-2 prevalence vary across space while age had a non-linear effect on HIV and HSV-2 prevalence. The posterior distribution was obtained by updating the prior distribution with the observed data. Since our study was fully Bayesian, inference was made by sampling from this posterior distribution. Markov Chain Monte Carlo (MCMC) is the most common estimation approach to inference for latent Gaussian models, however the method is slow and performs poorly when applied to such models [22]. The Integrated Nested Laplace (INLA) criterion, a relatively new technique developed to circumvent these shortfalls was used instead [22]. The SVC model was found to be better than the stationary model on the account of DIC.

The covariates used in these study had full information. This was obtained by deleting all missing values. More accurate results may be obtained by incorporating the weights to account for these deletion a task impossible for this study as the weights were based on different administrative units (provincial) instead of counties. The models introduced in this study can be replicated in other studies with similar data. Further work could be conducted to get the effect of the particular categories of the covariates e.g. for marital status, the effect of divorce, or single status e.t.c on each county. A comparison of this analysis with the recent KAIS 2012 data would reveal how the effects of the covariates in each region have changed over time and if the intervention strategies put in place have helped. Other models such as the simultaneous autoregressive model can be used in place of the conditional autoregressive model to relax the stationarity assumption. Since the CAR assumes normality, this assumption can be relaxed or we may altogether use a non-parametric approach.

### Data availability

The authors confirm that all data underlying the findings are fully available without restriction. The data is held by the Kenya National Bureau of Statistics and freely available to the public but a request has to be sent to the Kenya National Bureau of Statistics. The link to access it is http://statistics.knbs.or.ke/nada/index.php/catalog/25.

## Appendix 1

### The Random walk model

Random walk (RW) models can be used as priors to derive the discretized Bayesian smoothing spline estimator [32]. The Random walk was made spatially adaptive by introducing local smoothing parameters into the models [33, 34]. The random walk model of order 2 (RW2) for the Gaussian vector ***X*** = (*x*_*1*_, …, *x*_*n*_) is constructed assuming independent second-order increments:

$$ {\varDelta}^2{x}_i={x}_i\mathit{\hbox{-}} 2{x}_{i+ 1}+{x}_{i+ 2}\sim N\left( 0,{\tau}^{\mathit{\hbox{-}} 1}\right) $$

The density of ***X*** is derived from its n-2 s-order decrements as:

$$ \pi \left(X\left|\tau \right.\right)\mu {\tau}^{\left(n\mathit{\hbox{-}} 2\right)/ 2} exp\left\{\mathit{\hbox{-}}\frac{\tau }{2}{\displaystyle \sum {\left({\varDelta}^2{x}_i\right)}^2}\right\}={\tau}^{\left(n\mathit{\hbox{-}} 2\right)/ 2} exp\left\{\mathit{\hbox{-}}\frac{1}{2}{X}^TQX\right\} $$

The term *x*_*i*_ ‐ *2x*_*i* + *1*_ + *x*_*i* + *2*_ can be interpreted as an estimate of the second-order derivative of a continuous time function *x*(*t*) at *t* = *i* using the values of *x*(*t*) at *t* = *i*, *i* + 1, *i* + 2 [35]. The RW2 model is quite flexible due to its invariance to addition of a linear trend, and also computationally convenient due to its Markov properties i.e.

*π*(*x*_*i*_|*x*_− *i*_) = *π*(*x*_*i*_|*x*_*i* − 2_, *x*_*i* + 1_, *x*_*i* + 2_) for 2 < *i* < *n* − 2. RW2 is also a GMRF for which efficient numerical methods for sparse matrix in place of Markov chain Monte Carlo algorithms exists [36, 37].

## Appendix 2

### The Bayesian Spatially Varying Coefficient Process (BSVCP)

The specification of the BSVCP is in a hierarchical manner. The first stage is to specify the distribution of the data conditional on unknown parameters, and the second stage is specifying these unknown parameters conditional on other parameters.

The SVCP model is:

$$ {y}_{ijk}\left|{p}_{ijk}\sim Bernoulli\left({p}_{ijk}\right)\right.. $$

$$ h\left({p}_{ijk}\right)={\boldsymbol{X}}^{\boldsymbol{T}}{\boldsymbol{\beta}}_{\boldsymbol{k}}+{\boldsymbol{W}}^{\boldsymbol{T}}{\boldsymbol{\gamma}}_{\boldsymbol{k}} $$

The prior distribution for the regression coefficients is given as:

$$ \left[\boldsymbol{\upgamma} \left|{\boldsymbol{\mu}}_{\boldsymbol{\upgamma}},{\displaystyle {\sum}_{\boldsymbol{\upgamma}}}\right.\right]=\boldsymbol{N}\left({\boldsymbol{1}}_{\boldsymbol{n}\times \boldsymbol{1}}\otimes {\boldsymbol{\mu}}_{\boldsymbol{\upgamma}},{\displaystyle {\sum}_{\boldsymbol{\upgamma}}}\right) $$

[12]

Where:

${\boldsymbol{\mu}}_{\boldsymbol{\gamma}}={\left({\boldsymbol{\mu}}_{{\boldsymbol{\gamma}}_{\boldsymbol{0}}},{\boldsymbol{\mu}}_{{\boldsymbol{\gamma}}_{\boldsymbol{1}}},\dots, {\boldsymbol{\mu}}_{{\boldsymbol{\gamma}}_{\boldsymbol{p}}}\right)}^{\boldsymbol{T}}$ is the vector of means of the regression coefficients corresponding to each of *P* explanatory variables. Spatial dependence is taken into account through the covariance ∑_**γ**_. This is achieved by specifying the priors for **γ**^'^*s* as an aerial unit model e.g. the conditional autoregressive model (CAR) or the spatial autoregressive model (SAR) [38] or a geostatistical approach, where a parametric distance-based covariance function is specified [12]. Our focus is on the aerial unit model and in particular we assume the CAR priors for the ***γ***^'^***s***.

### Conditional autoregressive (CAR) Model

Consider a vector *ϕ* = (*ϕ*_1_, …, *ϕ*_*p*_)^*T*^ of *p* components that follows a multivariate Gaussian distribution with mean 0 and *B* as the inverse of the dispersion matrix, so that *B* is a *p* × *p* symmetric and positive definite matrix. The density for *ϕ* is given by:

$$ p\left(\phi \right)={\left(2\pi \right)}^{\frac{p}{2}}{\left|B\right|}^{\frac{1}{2}} \exp \left(-\frac{1}{2}{\phi}^TB\phi \right) $$

For the CAR model, the conditional distribution of a particular component given the remaining components is considered. In terms of the elements of the matrix *B* = (*b*_*ij*_), from the normal theory, *ϕ*_*i*_ has a full conditional distribution;

$$ p\left({\phi}_i\left|{\phi}_{-i}\right.\right)\propto \exp \left(-\frac{1}{2}{b}_{ii}{\left({\phi}_i-{\displaystyle \sum_{j\ne i}\frac{-{b}_{ij}}{b_{ii}}}{\phi}_j\right)}^2\right) $$

which is normally distributed i.e. ${\phi}_i\left|\Big|,{\phi}_{-i}\right.\sim N\left({\displaystyle \sum_{j\ne i}\frac{-{b}_{ij}}{b_{ii}}{\phi}_j,\frac{1}{b_{ii}}}\right)$ [39]

Mardia [40] showed the conditions under which the full conditional distributions specified above uniquely define a full joint distribution.

We let ${c}_{ij}=\frac{-{b}_{ij}}{b_{ii}}$ and ${b}_{ii}=\frac{1}{\sigma_i^2}$ and form a matrix *C* with ${c}_{ii}=0\; and\;{c}_{ij}=-\frac{-{b}_{ij}}{b_{ii}}$, and another matrix *M* = *Diag*(*σ*_*i*_^2^) and *M*^− 1^ = *Diag*(*b*_*ii*_)). The inverse of the dispersion matrix, **B** is then related to **C** and **M** as:

$$ B={M}^{-1}\left(I-C\right). $$

***I*** is the identity matrix and the joint distribution of *ϕ is MVN*(0, *M*^− 1^(*I* − *C*)). C and M must be modeled properly to ensure the symmetry of B, and this is achieved by conditioning *c*_*ij*_*σ*_*j*_^2^ = *c*_*ji*_*σ*_*i*_^2^. The C matrix is also specified to show relationship between neighbors. The elements of matrix C are defined as ${c}_{ii}=0\; and\;{c}_{ij}=\frac{1}{m_i}$ [39], if *j* is adjacent to *i* and zero otherwise. This is a commonly used adjacency matrix for lattice data. *m*_*i*_ represent the number of neighbors of region *i*. Define another matrix *W* to hold the adjacency structure, where, *w*_*ij*_ = 1 if region *i* and region *j* are neighbors and zero otherwise. Then, $C={W}_s\; where\;{W}_s= diag\left(\frac{1}{m_i}\right)W$. i.e. *W*_*s*_ is a scaled adjacency matrix, the *i*^*th*^ row being scaled by the number of neighbors of region *i*. The above expressions for the elements of C and M translate to the following specifications for inverse covariance matrix B: *b*_*ii*_ = *λm*_*i*_, *and b*_*ij*_ = − *λ* if *j* is adjacent to *i* and 0 otherwise. Thus B is symmetric and it can be expressed as *B* = *λ*(*Diag*(*m*_*i*_) − *C*). The expression *M*^− 1^(*I* − *C*) has a positive definite structure for the conditional distribution to give rise to a valid probability distribution function (pdf). The definition of the adjacency matrix above leads to an improper joint pdf. This is overcome by introducing a parameter *α* into the precision matrix B, to give:

$$ B={M}^{-1}\left(I-\alpha C\right) $$

If |*α*| < 1 then the matrix *M*^− 1^(*I* − *αC*) is diagonally dominant and symmetric. Symmetric and diagonally dominant matrices are positive definite [41].

## Additional file

Additional file 1: Text S1. R-INLA codes used in the analysis. (R 12 kb)
